# Clinical prediction models assessing response to radiotherapy for rectal cancer: protocol for a systematic review

**DOI:** 10.1186/s41512-022-00132-y

**Published:** 2022-10-06

**Authors:** Margarita Karageorgou, David M. Hughes, Arthur Sun Myint, D. Mark Pritchard, Laura J. Bonnett

**Affiliations:** 1 Department of Molecular and Clinical Cancer Medicine, Institute of Systems, Molecular and Integrative Biology, University of Liverpool, Liverpool, UK; 2 Department of Health Data Science, Institute of Population Health, University of Liverpool, Waterhouse Building, Block B, Brownlow Street, Liverpool, L69 3GF UK; 3 Papillon Suite, Clatterbridge Cancer Centre, Liverpool, UK

**Keywords:** Prediction, Risk factors, Radiotherapy, Rectal cancer, Meta-analysis

## Abstract

**Background:**

Rectal cancer has a high prevalence. The standard of care for management of localised disease involves major surgery and/or chemoradiotherapy, but these modalities are sometimes associated with mortality and morbidity. The notion of ‘watch and wait’ has therefore emerged and offers an organ-sparing approach to patients after administering a less invasive initial treatment, such as X-ray brachytherapy (Papillon technique). It is thus important to evaluate how likely patients are to respond to such therapies, to develop patient-tailored treatment pathways. We propose a systematic review to identify published clinical prediction models of the response of rectal cancer to treatment that includes radiotherapy and here present our protocol.

**Methods:**

Included studies will develop multivariable clinical prediction models which assess response to treatment and overall survival of adult patients who have been diagnosed with any stage of rectal cancer and have received radiotherapy treatment with curative intent. Cohort studies and randomised controlled trials will be included. The primary outcome will be the occurrence of salvage surgery at 1 year after treatment. Secondary outcomes include salavage surgery at at any reported time point, the predictive accuracy of models, the quality of the developed models and the feasibility of using the model in clinical practice.

Ovid MEDLINE, PubMed, Cochrane Library, EMBASE and CINAHL will be searched from inception to 24 February 2022. Keywords and phrases related to rectal cancer, radiotherapy and prediction models will be used. Studies will be selected once the deduplication, title, abstract and full-text screening process have been completed by two independent reviewers. The PRISMA-P checklist will be followed. A third reviewer will resolve any disagreement. The data extraction form will be pilot-tested using a representative 5% sample of the studies reviewed. The CHARMS checklist will be implemented. Risk of bias in each study will be assessed using the PROBAST tool. A narrative synthesis will be performed and if sufficient data are identified, meta-analysis will be undertaken as described in Debray et al.

**Discussion:**

This systematic review will identify factors that predict response to the treatment protocol. Any gaps for potential development of new clinical prediction models will be highlighted.

**Trial registration:**

CRD42022277704.

**Supplementary Information:**

The online version contains supplementary material available at 10.1186/s41512-022-00132-y.

## Background

According to the Association of Coloproctology of Great Britain and Ireland, rectal cancer is the third most common cancer in men and second in women in the United Kingdom (UK) [1]. The current guidelines for patients who do not have disseminated metastastic disease (cancer which has spread beyond the primary site) at the time of diagnosis recommend surgical treatment with or without chemo-radiotherapy (CRT) depending on the stage of the disease. An organ-sparing approach without surgery is also emerging as an effective option for some patients, based on effective CRT protocols and ‘watch-and-wait’ strategies with regular follow-up checks [2, 3]. Alongside this approach, active patient involvement in deciding their treatment options is also being promoted. As a result, patients who do not wish to have a permanent stoma (an artificial opening on the abdomen which connects the large bowel and allows waste, gas and faeces, to be diverted out of the body) are able to explore other possible treatment options for their disease [4].

One such option is contact X-ray brachytherapy, also known as contact radiotherapy (Papillon technique) which delivers a high dose of low energy X-rays straight onto the rectal tumour [5, 6]. It can be used in selected patients (often in addition to CRT) to treat rectal cancer and potentially avoid the need for surgery. According to the UK National Institute for Health and Care Excellence (NICE) guidelines, patients should not only be offered organ-sparing treatment options, but they also have the right to choose one of the approved treatments that best suits their needs [7]. In order to be able to make a choice, a patient needs to have the necessary background information and well-rounded knowledge of all the available options.

Clinical prediction models combine multiple pieces of patient information to make predictions about clinical outcomes in people who have an underlyimg condition. They can be used to inform patient counselling, guide treatment choice and stratify patients within clinical trials [8, 9]. Such a model, that is easy to understand and to use, may prove to be helpful to both clinicians and patients, especially now that new ‘watch-and-wait’ protocols for rectal cancer have emerged due to the extensive use of neo-adjuvant treatment (n-AT) [10]. A systematic review of risk prediction models in colorectal disease was undertaken in 2020 [11]. Although the review identified 24 risk prediction models and 51 risk factors that evaluate the colorectal tumour burden, none of these is widely used [11]. Additionally, no assessments of risk bias of the included models were undertaken. Consequently, an updated systematic review, targeting response to radiotherapy in people with rectal cancer, with an assessment of risk of bias, has the potential to minimise unnecessary operations and chemoradiotherapy side effects. Our review will highlight whether a suitable model already exists, whether an existing model can be updated in light of new evidence, or whether a new model should be developed.

We hope to create a new clinical prediction model, or update an existing one, for contact X-ray brachytherapy in rectal cancer and will therefore firstly systematically review existing clinical prediction models for the role of any type of radiotherapy in treating this disease. Our systematic review focuses on radiotherapy, with radical intent-treated rectal cancer patients. We will not include patients who have been treated for disease palliation.

### Research aims

The aim of this systematic review is to identify and summarise existing clinical prediction models and clinical decision rules predicting response to radiotherapy treatment, in adults with rectal cancer. The response to treatment will be assessed by the need of salvage surgery either immediately after treatment or later. According to the literature, the most common timepoint for salvage surgery occurs within the first year of radiotherapy treatment [12]. This review will identify and summarise studies of any prospective or restrospective design which utilise multiple prognostic factors in combination to predict the individualised risk of response.

## Methods

Overall, the PICO question is formulated [13]:

### Population

The study population are adult patients diagnosed with any stage of rectal cancer who have received radiotherapy as a component of their treatment regimen.

### Intervention

The study intervention is the radiotherapy for rectal cancer treatment administered with curative intent.

### Comparator

A comparator is not applicable to a systematic review of clinical prediction models. The comparator regarding the population type is patients with rectal cancer who in their treatment protocol did not receive any form of radiotherapy.

### Outcome(s)

The primary outcome is the assessment of overall survival of the patients and of the patient’s response to treatment (radiotherapy), i.e., the need for salvage surgery.

### Selection criteria

#### Study design

The review will include studies which have developed and/or validated or compared prediction models to predict the response of rectal cancer to radiotherapy. Types of studies to be included are randomised controlled trials and cohort studies which are either prospective or retrospective and combine multiple prognostic factors to predict the outcome. Case–control studies will be excluded.

#### Study population

This review will include adults from all sexes who have a diagnosis of rectal cancer (any tumour node metastasis (TNM) stage) in whom treatment protocol radiotherapy regimens were administered with curative intent. Patients who received radiotherapy for palliation will be excluded, as well as any non-rectal cancer primary site diagnosis. Studies with mixed populations, including those outside of the remit, will be included provided that the appropriate data for our defined group of patients is extractable.

Eligible prediction models will include patients who may respond to treatment and were thus recruited to the study at the time of treatment.

#### Setting

Studies in any setting will be included.

#### Potential prediction models

Studies must report a clinical prediction model utilising multiple prognostic factors to predict the chance of response to treatment following diagnosis of rectal cancer.

#### Study outcomes

This systematic review will evaluate the predictive accuracy of clinical prediction models identified in the literature to evaluate patient response to radiotherapy treatment for rectal cancer at 1 year, our primary outcome. This will be determined by the need for salvage surgery after treatment. An additional outcome of this study will be the predictive accuracy of models assessing the overall survival of patients through patient follow-up assessment. Additional secondary outcomes will consider the response at different time points, quality of the developed models in terms of the use of appropriate statistical methodology, and the feasibility of using the model in clinical practice.

#### Search strategy

Bibliographic databases will be searched for studies to include in the review. Ovid Technologies, Inc., part of the Wolters Kluwer group (Ovid MEDLINE) will be searched, PubMed free search engine will be used. The Cochrane Library (Wiley) will also be searched to identify any potentially similar systematic reviews. The HDAS (Healthcare Databases Advanced Search) platform will also be used to access EMBASE (Excerpta Medica database by Elsevier) and CINAHL (Cumulative Index to Nursing and Allied Health Literature) by EBSCO.

Searches will be performed using keywords and phrases related to rectal cancer, radiotherapy and prognostic models [13–16]. The full-search strategy is shown in Additional file 1.

References cited in identified sources will be examined to supplement the database searches. Sources of all languages and time periods will be searched. To identify other studies which have not yet been published, abstracts for conferences relevant to our research will be searched. Relevant systematic reviews will also be searched for further studies to include.

#### Study selection

Firstly, the search results from the databases will be deduplicated using reference manager Endnote X9, and then, the titles and abstracts will be searched, and if thought relevant, full texts will be identified and compared against the eligibility checklist. This will be performed by two independent reviewers (MK and DH) using pre-defined screening criteria and a full list of inclusion criteria. If any discrepancies cannot be resolved between the two reviewers, a third reviewer (LJB) will be sought for a solution [17, 18]. When required, additional information to ascertain eligibility will be requested from the study authors. If studies are not chosen for inclusion, the exclusion reason will be documented. A PRISMA-P (Preferred Reporting Items for Systematic Review and Meta-analysis Protocols) flow chart will be created and can be found in the Additional file 2 [19–21].

Non-English studies will be translated where necessary and possible to facilitate interpretation and data extraction.

#### Data extraction

Data will be extracted by two reviewers independently using an in-depth piloted data extraction form. Disagreements will be resolved through discussion or referral to a third reviewer. The data extraction form will be pilot tested using a representative 5% sample of the studies to be reviewed. Consensus between review authors will be gained before any modifications are made to the form. If major changes are needed after the first testing, the pilot testing will be repeated on a new set of 5% of the studies.

In terms of data extraction, study characteristics, study design characteristics, patient characteristics, candidate prognostic factors considered including the information on missing data, outcome measures, statistical methods employed and how prognostic factors included in the analysis were handled; and prediction model information including the method used in the final model will be extracted as will the prognostic factors used in the model. The data extraction specifically related to clinical prediction models will include the final model (its specification, included factors, values of regression coefficients and standard errors), how it was developed and any internal or external validation performance statistics for discrimination (such as the *c*-statistics or area under the curve) or for calibration (such as the expected/observed events ratio), together with their associated measures of spread [22]. This will be informed by the Critical Appraisal and Data Extraction for Systematic Reviews of Prediction Modelling Studies (CHARMS) checklist which helps frame the review question, design the review and extract the relevant items from the reports of the primary prediction modelling studies [23].

If deemed necessary, the author will be contacted to clarify any issues or in an attempt to retrieve any missing information.

#### Assessment of study quality

In this systematic review of prediction models in rectal cancer treatment, the assessment of bias and applicability to the intended population and setting will be performed using PROBAST (Prediction model Risk Of Bias Assessment Tool) [24]. This tool is intended to evaluate studies developing, validating or updating (for example, extending) prediction models, both diagnostic and prognostic. The study design and sample size will also be included in the risk of the bias assessment. Moreover, the reviewers will consider how missing data and continuous variables were handled, how the prognostic factors in the final model were chosen and whether there was any internal or external validation of the model [23].

#### Evidence synthesis

Any studies reporting the development of a prediction model will be summarised narratively, in particular what prognostic factors were included in the final model, how the included variables were coded, what the specification of the model was and how it produces an individual outcome probability or risk score, the reported predictive accuracy of the model and whether the model was validated internally and/or externally, and if so how.

If multiple studies are found that externally validate the same prediction model, then calibration statistics (such as expected/observed events) and discriminatory statistics (such as the *c*-statistic or area under the curve) will be synthesised using random-effects meta-analysis methodology of Debray and Snell to summarise the model’s average performance across different settings and its predicted performance in a future setting [25, 26]. If there are updated versions of the same prediction model identified in our review, then only statistics for the most recent model will be included in the meta-analysis.

If we identify multiple prediction models that have been adequately externally validated, we will compare their performance narratively, taking into account the different case mix, how this relates to our own setting, Clatterbridge Cancer Centre NHS Foundation Trust and also the quality of studies. The Clatterbridge Cancer Centre NHS Foundation Trust is a centre of excellence for contact X-ray brachytherapy treatment, namely Papillon technique, and receives referrals not only on a national but also on an international level. We have the largest cohort of adult patients treated for rectal cancer with this modality.

#### Analysis of subgroups or subsets

If there are sufficient relevant prediction models available, subgroup analyses will synthesise calibration and discrimination statistics for studies according to the setting of radiotherapy treatment implementation; i.e., neo-adjuvant or adjuvant conducted in different settings (countries) or different types of studies (prospective studies vs. randomised studies vs. randomised trails) or different model types (logistic vs. survival analysis) [27, 28].

## Discussion

The results of this systematic review will identify the factors predicting response to the treatment protocol. It will also identify all the currently available statistical models for prognosis and will provide insight into their applicability. Moreover, any gaps for potential development of new clinical prediction models will be highlighted.

Our results have the potential to inform the clinical management of patients diagnosed with rectal cancer. In particular, the results of the review will identify clinical prediction models for the response to treatment after diagnosis. These will be informative for clinicians currently treating patients and help inform treatment choices. The review will also identify areas where the evidence for or against particular candidate prediction models is lacking, and this will lead to recommendations for initiating additional prediction model development and validation.

## Supplementary Information

**Additional file 1.** Full search strategy tables.

**Additional file 2. **PRISMA-P 2015 Checklist.

## Data Availability

Not applicable.

## Abbreviations

UK: United Kingdom
CRT: Chemoradiotherapy
NICE: National Institute for Health and Care Excellence
PICO: Population/praticipants, intervention, comparator, outcome
C-index: Concordance index
ROC: Receiver operating characteristic curve
AUC: Area under the curve
TNM: Tumour, node, metastasis
MEDLINE: Medical Literature Analysis and Retrieval System Online
PubMed: Public MEDLINE
CHARMS: Critical appraisal and data extraction for systematic reviews of prediction modelling studies
PROBAST: Prediction model risk of bias assessment tool
PRISMA-P: Preferred reporting items for systematic review and meta-analysis protocols
CAST: Active monitoring of cancer as an alternative to surgery
