# Supplementary material for: Clinical prediction models assessing response to radiotherapy for rectal cancer: protocol for a systematic review
Source: Diagn Progn Res. 2022 Oct 6;6:19. doi: 10.1186/s41512-022-00132-y (PMC9535989; doi:10.1186/s41512-022-00132-y)
Supplement: Supplementary file 1 — Additional file 1. Full search strategy tables. [file 41512_2022_132_MOESM1_ESM.docx]

Full Search strategy tables

| **Ovid MEDLINE(R) ALL <1946 to February 24, 2022>** | |
| --- | --- |
| 1 | Predict*.ti,ab. |
| 2 | exp "Predictive Value of Tests"/ |
| 3 | Scor*.ti,ab. |
| 4 | Observ*.ti,ab. |
| 5 | exp Observer Variation/ |
| 6 | 1 or 2 or 3 or 4 or 5 |
| 7 | "rectal cancer*".ti,ab. |
| 8 | (cancer adj3 rectum).ab,ti. |
| 9 | rectal cancer.mp. or exp Rectal Neoplasms/ |
| 10 | 7 or 8 or 9 |
| 11 | brachytherapy.mp. or exp Brachytherapy/ |
| 12 | radiotherapy.mp. or Radiotherapy, Adjuvant/ or exp Radiotherapy/ |
| 13 | radiotherapy.ti,ab. |
| 14 | brachytherapy.ti,ab. |
| 15 | 11 or 12 or 13 or 14 |
| 16 | 6 and 10 and 15 |

| **PubMed** | |
| --- | --- |
| Search number | Query Filters |
| 4 | ((#1) AND (#2)) AND (#3) |
| 3 | (((((rectal cancer*[Title/Abstract]) OR (rectal adenocarcinoma*[Title/Abstract])) OR (rectal neoplasm*[MeSH Terms])) OR (cancer, rectum[MeSH Terms])) OR (neoplasm, rectum[MeSH Terms])) OR (neoplasms, rectal[Title/Abstract]) |
| 2 | ((((radiotherap*[Title/Abstract]) OR (radiotherap*[MeSH Terms])) OR (brachytherapy[MeSH Terms])) OR (chemoradi*[Title/Abstract])) OR (radiation[MeSH Terms]) |
| 1 | (Predict*[tiab] OR Predictive value of tests[mh] OR Scor*[tiab] OR Observ*[tiab] OR Observer variation[mh]) |

| **COCHRANE SEARCH STRATEGY** |
| --- |
| Rectal adenocarcinoma OR rectal cancer OR rectal malignancy OR rectal carcinoma OR rectal neoplasm OR neoplasm of the rectum OR malignancy of the rectum OR cancer of the rectum OR adenocarcinoma of the rectum OR tumour of the rectum in All Text |
| AND |
| brachytherapy OR radiation OR radiotherapy OR chemoradiotherapy in All Text |
| AND |
| clinical prediction model OR clinical prediction rule OR clinical prognostic model OR prognostic factors OR prediction factors OR "predictive value of tests" OR "observation variance" in All Text |
| (Word variations have been searched) |
| Cochrane Reviews  Cochrane Protocols  Trials  Editorials/ Special Collections/ Clinical Answers |

| **EMBASE** | | |
| --- | --- | --- |
| 1 | [EMBASE](https://hdas.nice.org.uk/strategy/1132263/1/#EMBASE-panel) | "RECTUM CANCER"/ OR "RECTUM TUMOR"/ OR "RECTUM CARCINOMA"/ |
| 2 | [EMBASE](https://hdas.nice.org.uk/strategy/1132263/2/#EMBASE-panel) | (rectal cancer*).ti,ab |
| 3 | [EMBASE](https://hdas.nice.org.uk/strategy/1132263/3/#EMBASE-panel) | (rectal tumo*).ti,ab |
| 4 | [EMBASE](https://hdas.nice.org.uk/strategy/1132263/4/#EMBASE-panel) | *(1 OR 2 OR 3)* |
| 5 | [EMBASE](https://hdas.nice.org.uk/strategy/1132263/5/#EMBASE-panel) | "CANCER THERAPY"/ OR "CANCER RADIOTHERAPY"/ OR "ADJUVANT RADIOTHERAPY"/ OR CHEMORADIOTHERAPY/ OR "CANCER RECURRENCE"/ OR "CANCER REGRESSION"/ |
| 6 | [EMBASE](https://hdas.nice.org.uk/strategy/1132263/6/#EMBASE-panel) | (brachytherapy).ti,ab |
| 7 | [EMBASE](https://hdas.nice.org.uk/strategy/1132263/7/#EMBASE-panel) | (radiotherapy).ti,ab |
| 8 | [EMBASE](https://hdas.nice.org.uk/strategy/1132263/8/#EMBASE-panel) | *(5 OR 6 OR 7)* |
| 9 | [EMBASE](https://hdas.nice.org.uk/strategy/1132263/9/#EMBASE-panel) | "PREDICTOR VARIABLE"/ |
| 10 | [EMBASE](https://hdas.nice.org.uk/strategy/1132263/10/#EMBASE-panel) | "PREDICTIVE VALUE OF TESTS"/ |
| 11 | [EMBASE](https://hdas.nice.org.uk/strategy/1132263/11/#EMBASE-panel) | exp "PREDICTION AND FORECASTING"/ |
| 12 | [EMBASE](https://hdas.nice.org.uk/strategy/1132263/12/#EMBASE-panel) | "PREDICTIVE VALUE"/ |
| 13 | [EMBASE](https://hdas.nice.org.uk/strategy/1132263/13/#EMBASE-panel) | exp "OBSERVER VARIATION"/ |
| 14 | [EMBASE](https://hdas.nice.org.uk/strategy/1132263/14/#EMBASE-panel) | (pr* model).ti,ab |
| 15 | [EMBASE](https://hdas.nice.org.uk/strategy/1132263/15/#EMBASE-panel) | *(9 OR 10 OR 11 OR 12 OR 13 OR 14)* |
| 16 | [EMBASE](https://hdas.nice.org.uk/strategy/1132263/16/#EMBASE-panel) | *(4 AND 8 AND 15)* |

| **CINAHL** | | |
| --- | --- | --- |
| 1 | [CINAHL](https://hdas.nice.org.uk/strategy/1132280/1#CINAHL-panel) | (predict*).ti,ab |
| 2 | [CINAHL](https://hdas.nice.org.uk/strategy/1132280/2#CINAHL-panel) | (scor*).ti,ab |
| 3 | [CINAHL](https://hdas.nice.org.uk/strategy/1132280/3#CINAHL-panel) | (observ* variation).ti,ab |
| 4 | [CINAHL](https://hdas.nice.org.uk/strategy/1132280/4#CINAHL-panel) | exp PROGNOSIS/ OR exp "TREATMENT OUTCOMES"/ OR exp "CLINICAL ASSESSMENT TOOLS"/ OR exp "NEOPLASM GRADING"/ OR exp "NEOPLASM STAGING"/ OR exp "RISK ASSESSMENT"/ OR exp "SENSITIVITY AND SPECIFICITY"/ |
| 5 | [CINAHL](https://hdas.nice.org.uk/strategy/1132280/5#CINAHL-panel) | exp "MODELS, STATISTICAL"/ OR exp "PREDICTION MODELS"/ OR exp "ROC CURVE"/ OR exp "STATISTICS, TYPES"/ |
| 6 | [CINAHL](https://hdas.nice.org.uk/strategy/1132280/6#CINAHL-panel) | exp "PREDICTIVE VALUE OF TESTS"/ |
| 7 | [CINAHL](https://hdas.nice.org.uk/strategy/1132280/7#CINAHL-panel) | *(1 OR 2 OR 3 OR 4 OR 5 OR 6)* |
| 8 | [CINAHL](https://hdas.nice.org.uk/strategy/1132280/8#CINAHL-panel) | exp "RECTAL NEOPLASMS"/ |
| 9 | [CINAHL](https://hdas.nice.org.uk/strategy/1132280/9#CINAHL-panel) | "RECTAL DISEASES"/ |
| 10 | [CINAHL](https://hdas.nice.org.uk/strategy/1132280/10#CINAHL-panel) | (rectal adenocarcinoma*).ti,ab |
| 11 | [CINAHL](https://hdas.nice.org.uk/strategy/1132280/11#CINAHL-panel) | (rectal cancer*).ti,ab |
| 12 | [CINAHL](https://hdas.nice.org.uk/strategy/1132280/12#CINAHL-panel) | exp RECTUM/ |
| 13 | [CINAHL](https://hdas.nice.org.uk/strategy/1132280/13#CINAHL-panel) | *(8 OR 9 OR 10 OR 11 OR 12)* |
| 14 | [CINAHL](https://hdas.nice.org.uk/strategy/1132280/14#CINAHL-panel) | exp RADIOTHERAPY/ OR exp BRACHYTHERAPY/ OR exp CHEMORADIOTHERAPY/ OR exp "RADIOTHERAPY, ADJUVANT"/ |
| 15 | [CINAHL](https://hdas.nice.org.uk/strategy/1132280/15#CINAHL-panel) | (radiotherapy).ti,ab |
| 16 | [CINAHL](https://hdas.nice.org.uk/strategy/1132280/16#CINAHL-panel) | (brachytherapy).ti,ab |
| 17 | [CINAHL](https://hdas.nice.org.uk/strategy/1132280/17#CINAHL-panel) | *(14 OR 15 OR 16)* |
| 18 | [CINAHL](https://hdas.nice.org.uk/strategy/1132280/18#CINAHL-panel) | *(7 AND 13 AND 17)* |
